# Effectiveness, efficiency and adverse effects of using direct or indirect bonding technique in orthodontic patients: a systematic review and meta-analysis

**DOI:** 10.1186/s12903-019-0831-4

**Published:** 2019-07-08

**Authors:** Yanxi Li, Li Mei, Jieya Wei, Xinyu Yan, Xu Zhang, Wei Zheng, Yu Li

**Affiliations:** 1 State Key Laboratory of Oral Diseases & National Clinical Research Center for Oral Diseases & Department of Oral Implantology, West China Hospital of Stomatology, Sichuan University, Chengdu, China; 2 Discipline of Orthodontics, Department of Oral Sciences, Sir John Walsh Research Institute, Faculty of Dentistry, University of Otago, Dunedin, New Zealand; 3 State Key Laboratory of Oral Diseases & National Clinical Research Center for Oral Diseases & Department of Conservative and Endodontic Dentistry, West China Hospital of Stomatology, Sichuan University, Chengdu, China; 4 State Key Laboratory of Oral Diseases & National Clinical Research Center for Oral Diseases & Department of Orthodontics, West China Hospital of Stomatology, Sichuan University, 14 Renmin South Road Third Section, Chengdu, 610041 China; 5 State Key Laboratory of Oral Diseases & National Clinical Research Center for Oral Diseases & Department of Oral and Maxillofacial Surgery, West China Hospital of Stomatology, Sichuan University, 14 Renmin South Road Third Section, Chengdu, 610041 China

**Keywords:** Dental bonding, Orthodontic brackets, Direct bonding, Indirect bonding

## Abstract

**Background:**

The direct and indirect bonding techniques are commonly used in orthodontic treatment. The differences of the two techniques deserve evidence-based study.

**Materials and methods:**

Randomized controlled trials (RCTs), wherein direct and indirect bonding techniques were used in orthodontic patients were considered. The MEDLINE, EMBASE, CENTRAL and Web of Science databases were searched to identify relevant articles published up to December 2018. Grey literature was also searched. Two authors performed data extraction independently and in duplicate using the data collection form. The included trials were assessed using the Cochrane risk of bias assessment tool.

**Results:**

Of the 1557 studies screened, 42 full articles were scrutinized and assessed for eligibility. Eight RCTs (247 participants) were finally included for the analyses. The qualitative synthesis showed that no significant difference existed in the accuracy of bracket placement and oral hygiene status between the two bonding techniques. The indirect bonding was found to involve less chairside time but more total working time compared with the direct bonding. The meta-analysis on bond failure rate demonstrated no significant difference between the direct and indirect bonding (RR = 1.13, 95% CI = 0.78–1.64, I^2^ = 22%, *P* = 0.50). Consistent results were obtained in the subgroup analyses and sensitivity analyses.

**Conclusion:**

Weak evidence suggested that the direct and indirect bonding techniques had no significant difference in bracket placement accuracy, oral hygiene status and bond failure rate, for bonding orthodontic brackets. The indirect bonding might require less chairside time but more total working time in comparison with the direct bonding technique. High-quality well-designed randomized controlled trials are needed before a conclusive recommendation could be made.

**Electronic supplementary material:**

The online version of this article (10.1186/s12903-019-0831-4) contains supplementary material, which is available to authorized users.

## Background

Direct bonding technique is commonly used for bonding brackets in orthodontic clinics [1, 2]. The indirect bonding technique was first proposed in 1972 for improving the accuracy of orthodontic bracket positioning [3, 4]. The latter mainly includes two stages, i.e. the laboratory stage and the clinical stage. Each bracket is precisely located on the study model during the laboratory stage; all the brackets are placed on the enamel integrally with the help of a tray during the clinical stage [5, 6].

Effectiveness (bracket placement accuracy), efficiency (total working time and chairside time) and adverse effects (oral hygiene status and bond failure rate) of the two techniques have been traced since they were proposed. The accuracy of bracket placement is of great clinical importance, especially for the pre-adjusted appliances [7]. Misplacement of orthodontic brackets could cause unwanted tooth movement, such as deviations in rotation, tipping, in/out, extrusion/intrusion, and torque [8]. Indirect bonding usually provides good vision and enough time to place brackets on the models, which facilitates the placement to some degree. But uncertainty remains on whether it acquires higher placement accuracy than direct bonding does for clinical treatment. Oral hygiene measure is usually significantly impeded by the fixed appliances used in orthodontic treatment [9]. Biofilm has been found to form around the bracket-adhesive-enamel junction [10, 11]. As a result, white spot lesions become a common problem in orthodontic patients. The excessive adhesive and its polymerization shrinkage promote plaque accumulation [10, 11]. Although it is difficult to completely remove the excessive adhesives in both the direct and the indirect bonding [11], indirect bonding seems to reduce the excessive adhesives [12]. Whether the two bonding techniques result in different oral hygiene status remains a question. Bond failure, the accidental detachment of a bracket during the orthodontic treatment [13], can significantly compromise the clinical efficiency [14], treatment result [15], patient’s satisfaction [16] and treatment confidence [17]. The bond failure has been found to be associated with bonding technique (direct/indirect) [18].

The aim of the study was to systematically review and compare the effectiveness (bracket placement accuracy), efficiency (total working time and chairside time) and adverse effects (oral hygiene status and bond failure rate) of direct and indirect bonding techniques in orthodontic patients.

## Methods

This study is not registered under any organization. This study followed the Preferred Reporting Items for Systematic Reviews and Meta-Analyses (PRISMA) statement guidelines (www.prisma-statement.org) [19, 20]. The checklist is shown as Additional file 3: Table S1.

### Search strategy and databases

A systematic search to identify all the relevant randomized controlled trials was conducted in the databases of MEDLINE (via PubMed), EMBASE, CENTRAL (The Cochrane Library), and Web of Science. No restrictions were employed on language or year of publication. A supplemental manual search was performed by reviewing the reference lists of the related articles. The search strategy was developed for MEDLINE and adapted for the other databases (Table 1). Grey literature was searched on Clinicaltrial.gov, OpenGrey and the World Health Organization’s International Clinical Trial Registry Platform. All searches were conducted on 24 July 2018, and updated on 14 December 2018.

**Table 1:** Search strategies used in the study

Database	Step	Keywords
MEDLINE	1	direct bonding
	2	indirect bonding
	3	“Dental Bonding/methods”[Mesh]
	4	“Orthodontic Brackets”[Mesh]
	5	1 or 2 or 3
	6	4 and 5
EMBASE	1	direct bonding.mp.
	2	indirect bonding.mp.
	3	exp dental bonding/
	4	exp orthodontic bracket/
	5	1 or 2 or 3
	6	4 and 5
CENTRAL	1	direct bonding: ti,ab,kw (Word variations have been searched)
	2	indirect bonding: ti, ab, kw (Word variations have been searched)
	3	MeSH descriptor: [Dental Bonding] explode all trees
	4	MeSH descriptor: [Orthodontic Brackets] explode all trees
	5	#1 or #2 or #3
	6	#4 and #5
Web of Science	1	TS = *direct* bond*
	2	TS = orthodonti*
	3	TS = bracket*
	4	#1 AND #2 AND #3

### Selection criteria

The following inclusion criteria were applied: (1) Study design: randomized controlled trials (RCTs); (2) Participants: patients requiring orthodontic treatment using bonding technique; (3) Intervention and control: direct and indirect bonding techniques for bonding orthodontic brackets. (4) Outcomes: bracket placement accuracy, total working time, chairside time, oral hygiene status, bond failure rate. No restrictions were implemented regarding the adhesive and bracket. The exclusion criteria of the study included case-control studies, cross-sectional studies, case reports, in vitro studies, reviews, conference abstracts and letters, as well as studies in which subjects had systematic diseases.

### Data extraction and analysis

Two reviewers (Y.X.L. and J.W.) screened the titles and abstracts of the identified studies independently and in duplicate. The Kappa statistics was used to test the interrater reliability, with a larger value corresponding to a greater reliability (i.e. 0, 0.2, 0.4, 0.6, 0.8 and 1.0 indicating none, slight, fair, moderate, substantial and perfect agreement, respectively). Briefly, the number of studies included/excluded through screening the titles and abstracts by each reviewer was tabulated and compared using Cohen’s Kappa; the number of studies included/excluded through assessing the full articles was also compared using Cohen’s Kappa. Studies not meeting the inclusion criteria were excluded, and the reasons for exclusion were noted (Additional file 4: Table S2). The two reviewers (Y.X.L. and J.W.) independently extracted data from the studies using a data extraction form. The following data were collected: author and year of publication, study design, observation period, number and age of participants, inclusion criteria, details of intervention and control, as well as outcomes. Consensus was obtained by discussion with the third reviewer (L.M.) to resolve any disagreements during study selection and data extraction.

### Methodological quality assessment

Each RCT was assessed using the evaluation method recommended by the Cochrane Hand- book for Systematic Reviews for Interventions 5.1.0 (http://handbook.cochrane.org). Two reviewers (Y.X.L. and J.W.) appraised the studies independently according to the following aspects: random sequence generation, allocation concealment, blinding, completeness of outcome data, selective outcome reporting, and other biases. Each aspect was classified as having either a low, high, or unclear risk of bias. The Kappa statistics was calculated to test interrater reliability. The overall level of risk for each study was subsequently classified as low (all quality items were met), unclear (unclear risk of bias for one or more domain), or high (high risk of bias for one or more domain).

### Statistical analysis

Quantitative analysis was performed using Review Manager 5 (version 5.3; Nordic Cochrane Centre, Cochrane Collaboration, Copenhagen, Denmark). For dichotomous data, the risk ratio (RR) and 95% confidence interval (95% CI) for each study were calculated. I^2^ index served as an indicator of true heterogeneity in percentages in the study, with a larger value corresponding to a greater heterogeneity (i.e. 25, 50 and 75% indicating a low, moderate and high heterogeneity, respectively). The random-effects model was applied. The statistical significance for the hypothesis test was set at *P* < 0.05 (2-tailed Z-test). Subgroup analyses were performed based on the age of participants and adhesive types. Sensitivity analyses were conducted to test the stability of the results of the meta-analyses. Funnel plot would be generated to assess publication bias when more than 10 studies were included [21–23].

## Result

**Fig. 1:**
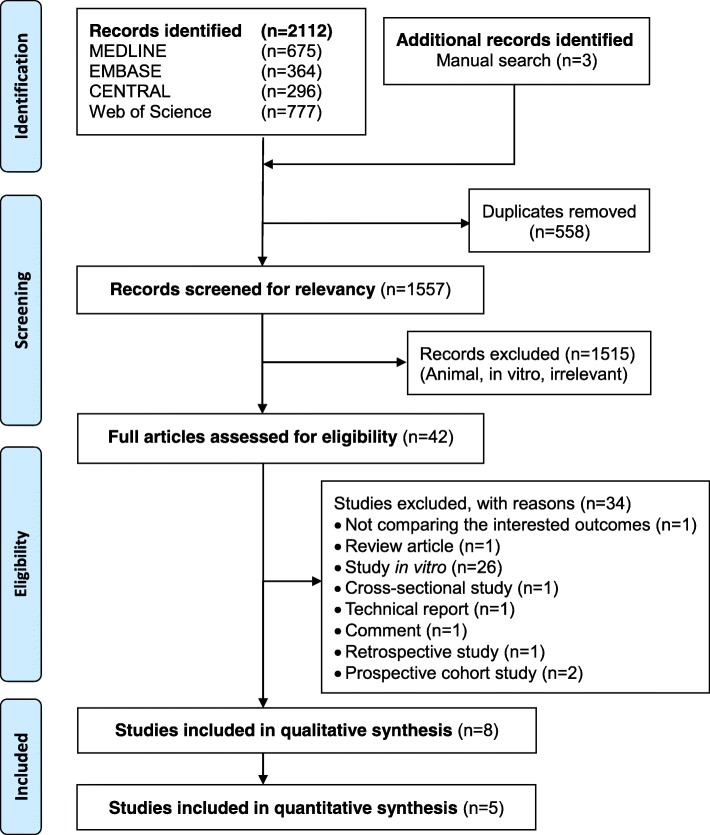
Study flow chart of the systematic review

There was a moderate agreement between the two observers’ judgment for screening studies (K = 0.729, 95% CI, 0.590, 0.846, *p* < 0.001) and a perfect agreement for including studies in this systematic review (K = 1.000, 95% CI, 1.000, 1.000, p < 0.001). The study flowchart is shown in Fig. 1. The initial search from all sources yielded 2115 records. After screening the titles and abstracts, 2073 records were excluded (558 duplicated records and 1515 records unrelated to this systematic review). As a result, 42 articles remained for full-text assessment, and based on the predetermined eligibility criteria, 34 articles were excluded (Additional file 4: Table S2). Finally, eight studies (247 participants with brackets) were included in the systematic review [7, 12, 18, 24–28].

### Characteristics of the included studies

The basic characteristics of the eight RCTs included in the study are summarized in Table 2. The orthodontic brackets that were used in the included studies were all non-customised labial/buccal brackets. All the recruited studies used a split-mouth design except for the studies of Yıldırım et al. [27] and Huang et al. [28].

**Table 2:** Summary of the included studies

Study	Study design	Observation period	Participants	Intervention (Indirect bonding adhesives)	Comparison/Control (Direct bonding adhesives)	Outcomes		
						Outcomes	Indirect	Direct
Aguirre 1982 [24]	RCT (split-mouth)	3 months	Sex: not specified Age: patients from graduate practice Inclusion criteria: receiving the orthodontic appliance Brackets: Ormco standard, with a mesh pad	Endur (self-curing) (Lab adhesive: Sugar Daddy Tray: Citricon silicone impression material)	Not specified	Bracket placement accuracy	refer to the original data	
						Working time (min, mean ± SD)	LS: 29.8 ± 0.8 CS: 23.7 ± 1.1 T: 53. 5	42.0 ± 0.9
						Bond failure rate	4/98 (4.10%)	5/94 (5.30%)
Dalessandri 2012 [12]	RCT (split-mouth)	2 years and 6 months ±4 months	Sex: male 13, female 17 Age: 11.2–12.8 years Inclusion criteria: attending an orthodontics department of a university and undergoing fixed orthodontic treatment (Victory braces, Low Profile series, MBT prescription, 0.022-in. slot, power arm on canines, 3 M Unitek); no presence of systemic or local (caries, periodontal pockets, mucosal alterations) pathologies; no need for surgical or extended restorative adjunctive procedures; no enamel development alterations; no dietary restrictions or intolerances Brackets: Victory braces, Low Profile series, MBT prescription, 0.022-in. slot, power arm on canines	3 M Unitek (light-cured) (Lab adhesive: removable adhesive (Vinavil, Vinavil SpA) Tray: a first bracket transfer matrix in a soft material (Copyplast 0.5 mm, Scheu Dental GmbH) was prepared with a pressure molding machine (Biostar VI, Scheu Dental Technology). and a second transfer matrix with a 1-mm thick Duran (Scheu Dental GmbH) foil was developed)	3 M Unitek (light-cured)	Oral hygiene status (number of new caries)	2	2
						Oral hygiene status (plaque accumulation index)	0.97 ± 0.29	1.71 ± 0.97
						Oral hygiene status (number of new white spots)	8	21
Hodge 2004 [7]	RCT (split-mouth)	Not specified	Sex: not specified Age: not specified Inclusion criteria: requiring full treatment with preadjusted edgewise appliances in both arches Brackets: not specified	Not specified	Not specified	Bracket placement accuracy (bracket placement errors; mm/°, mean ± SD)	V: −0.20 ± 0.08 H: −0.05 ± 0.10 A: 0.02 ± 0.05	V: −0.27 ± 0.46 H: −0.11 ± 0.30 A: 0.08 ± 0.14
Huang 2016 [28]	RCT	Not specified	Sex: male 12, female 33 Age:15–30 years Inclusion criterion: requiring fixed orthodontic appliance therapy; with good oral hygiene; no periodontal diseases, caries, fluorosis, cracked-teeth, and partially erupted cases etc., heights of clinical crowns were similar Brackets: Damon3MX self-ligating brackets (Ormco, US)	3 M Unitek Sondhi™ Rapid-set (3 M, US) (Lab adhesive: 3 M Unitek Transbond™ XT (3 M, US) (light-cured) Tray: using translucent soft silicone to make the soft layer and rigid tray refinement)	3 M Unitek Transbond™ XT (3 M, US) (light-cured)	Working time (min, mean)	LS: 56.4 CS: 34.13 T: 90.53	T: 43.5
Thiyagarajah 2006 [25]	RCT (split-mouth)	12 months	Sex: not specified Age: 12–15 (mean13.6) years Inclusion criteria: requiring orthodontic treatment with full upper and lower pre-adjusted edgewise appliances; no caries, large restorations, fluorosis, hypoplasia or abnormalities of crown morphology Brackets: MBT™ Versatile+ Bracket System	Transbond TM XT (light-cured) (Lab adhesive: 3 M Unitek laboratory adhesive; Tray: 0.45 mm Drufolen WTM transparent tray)	Transbond TM XT (light-cured)	Bond failure rate	6/279 (2.15%)	8/274 (2.92%)
Vijayakumar 2014 [26]	RCT (split-mouth)	6 months	Sex: male 12, female 18 Age: 15–28 (mean 21.73) years Inclusion criteria: requiring fixed orthodontic appliance therapy; with full complement of tooth and good oral hygiene; no deep-bite, crowns and veneers, and partially erupted cases Brackets: MBT −022 brackets	Transbond XT (light cure adhesive) (Lab adhesive: Transbond XT light cure adhesive; Tray: constructed using soft clear thermo-plastic sheet of 2 mm thickness in a bio-star machine)	Transbond XT (light-cured)	Bond failure rate	23/262 (8.78%)	27/256 (10.55%)
Yıldırım 2018 [27]	RCT	Direct bonding: 12.0 ± 3.1 months; indirect bonding: 11.4 ± 2.4 months	Sex: male 7, female 23 Age: Direct: 11.7–19.2 (14.6 ± 2.4) years; indirect: 11.4–21(16.7 ± 5.1) years Inclusion criterion: complete permanent dentition, including second molars with bilateral Angle Class I molar and canine relationships, no previous orthodontic treatment, no skeletal discrepancy, mild or moderate crowding Brackets: 0.018-in slot stainless steel Roth prescription Empower 2 self-ligating brackets and 0.018-in Non Convertible LP direct bond molar tubes, American Orthodontics, Sheboygan, Wis	Transbond Supreme LV Low Viscosity Light Cure Adhesive; 3 M Unitek (light-cured) (Lab adhesive: Transbond Plus Color Change Adhesive; Tray: 2-layer transfer tray prepared using translucent soft silicone [Memosil 2; Heraeus Kulzer] and thermoformed rigid Essix plastic [Raintree Essix, New Orleans, La])	Transbond Plus Color Change Adhesive; 3 M Unitek (light-cured)	Working time (min, mean ± SD)	LS: 45.54 ± 4.33 CS: 26.51 ± 3.33 T: 72.05 ± 5.2	T: 53.02 ± 4.72
						Oral hygiene status (plaque accumulation index; median ± interquartile range)	11.67 ± 16.66	5 ± 10.00
						Oral hygiene status (white spot lesions formation; median ± interquartile range)	0.00 ± 0.08	0.00 ± 0.16
						Bond failure rate	45/420 (10.71%)	30/420 (7.14%)
Zachrisson 1978 [18]	RCT (split-mouth)	6 months	Sex: male14, female 28 Age: 11–15 (13 ± 1.5) years Inclusion criteria: at least 4 permanent teeth in a quadrant Bracket: metal brackets with mesh-backed bases	Edur (self-curing) (Lab adhesive: Sugar Daddy; Tray: Optosil, silicone putty impression material)	Edur (self-curing)	Oral hygiene status (number of gingival index score)	0:24 1:1235 2:154	0:39 1:1556 2:148
						Oral hygiene status (number of plaque index score along bracket)	0:658 1:635 2:78	0:983 1:685 2:72
						Oral hygiene status (number of plaque index score along gingiva)	0:517 1:717 2:164	0:755 1:818 2:167
						Bond failure rate	9/104 (8.65%)	5/121 (4.13%)

*RCT* randomized controlled trial, *LS* laboratorial stage, *CS* clinical stage, *T* total, *V* vertical, *H* horizontal, *A* angular

Though the bond failure rate in the study by Zachrisson et al [18] involved several different metal brackets and adhesives, only data with comparability and homogeneity (i.e. the same mesh-backed metal brackets bonded with the same adhesive of Endur) in both direct and indirect bonding groups were included in this study.

### Risk of bias

**Fig. 2:**
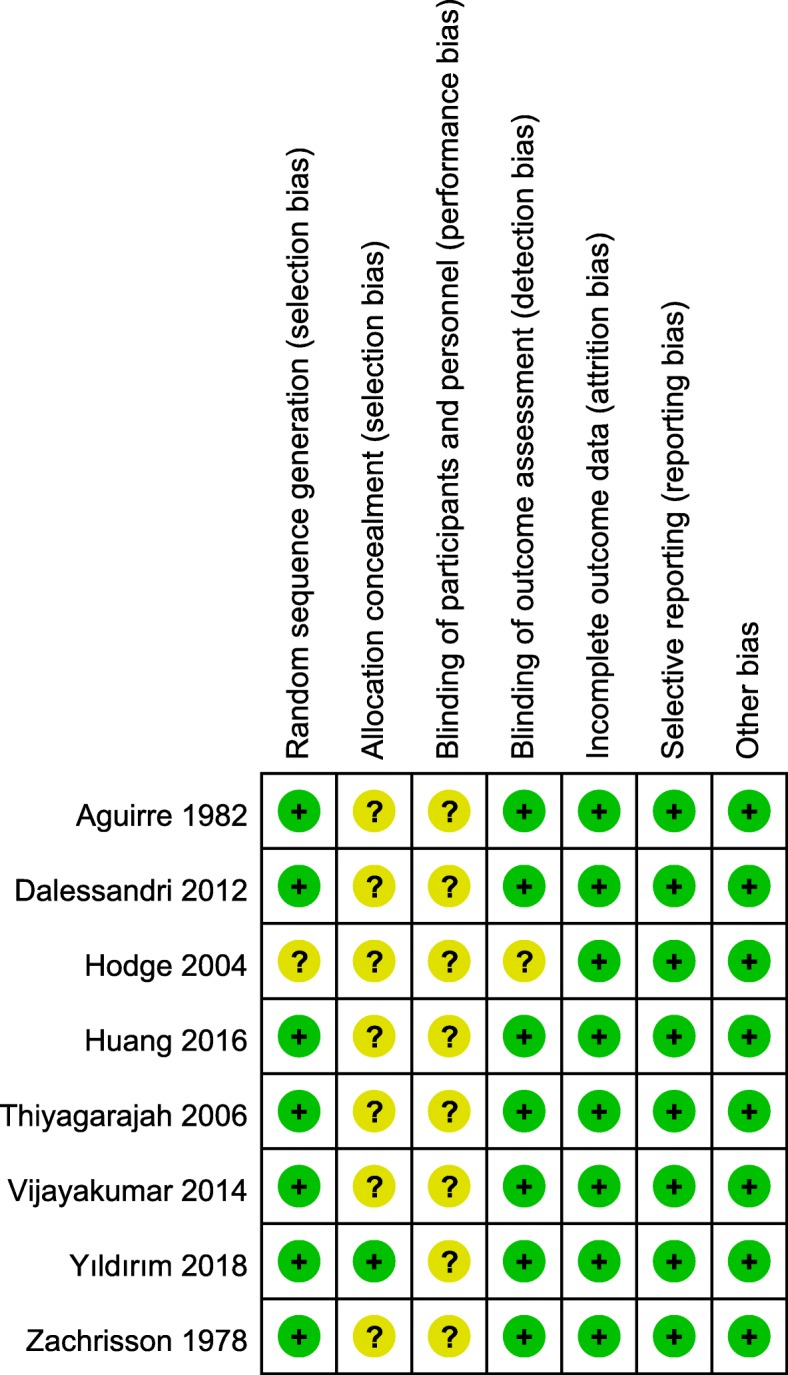
Risk of bias summary

**Fig. 3:**
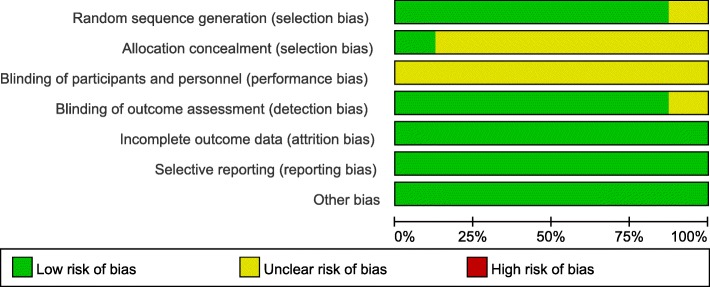
Risk of bias graph

There was a perfect agreement between the two observers’ assessment of included studies’ methodological quality (K = 0.968, 95% CI, 0.920, 1.000, *p* < 0.001). The risk of bias of the included studies [7, 12, 18, 24–28] was rated as unclear using the Cochrane Collaboration risk of bias tool (Figs. 2, 3 and Additional file 5: Table S3).

### Results of bracket placement accuracy

Two RCTs [7, 24] found no significant difference in bracket placement accuracy between the direct and indirect bonding. One study [7], in which the incisors and canines and the vertical, horizontal and angular errors were included and analyzed, suggested that the bracket placement error ranges were much smaller in the indirect bonding than that in the direct bonding. Another study [24] included the incisor, canines and premolars but only reported the vertical and angular errors. It found a statistically significant difference in bracket placement on certain teeth (i.e. the indirect bonding technique acquired higher vertical accuracy of bracket placement on maxillary canines and higher angulation accuracy on maxillary and mandibular canines; the direct bonding technique gained superiority on the angulation accuracy on mandibular second premolars). Meta-analysis of the bracket placement accuracy was not performed due to the methodological differences and extensive heterogeneity.

### Results of treatment efficiency

Three RCTs [24, 27, 28] compared the total working time (time spent on laboratorial and clinical stages) between the direct and indirect bonding techniques (the specific information is shown in Table 2). One study [24] reported operation time in hemi-arches in both jaws, while the other two studies [27, 28] reported the operation time in total-arches. All these three studies [24, 27, 28] found that the indirect bonding required significantly longer total working time and shorter clinical chairside time compared with the direct bonding. Meta-analysis of the treatment efficiency was not performed due to the methodological differences and extensive heterogeneity.

### Results of oral hygiene status

Three RCTs [12, 18, 27] compared the oral hygiene status between the direct and indirect bonding. Two studies [18, 27] found no significant difference in plaque accumulation around brackets, the formation of white spot lesions [27] and the gingival condition [18]. However, one study [12] revealed less plaque accumulation in the indirect bonding group than that in the direct bonding group during the first four months after brackets placement and later onset of white spots during the treatment. But plaque accumulation did not differ significantly considering whole-mouth results. No quantitative synthesis on the outcome of oral hygiene was performed due to the methodological differences and extensive heterogeneity.

### Results of bond failure rate

**Fig. 4:**
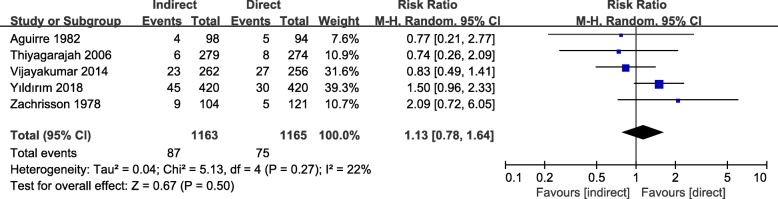
Forest plot for the risk ratio of bond failure rate comparing the direct and indirect bonding

Five RCTs [18, 24–27] compared the bond failure rate of direct and indirect bonding (Fig. 4). Meta-analysis was performed. Low heterogeneity was observed (I^2^  =  22%).

No difference of bond failure rate was found between the direct and indirect bonding (RR = 1.13, 95% CI = 0.78–1.64, I^2^ = 22%, *P* = 0.50). Subgroup analyses (Additional file 1: Figure S1) on the age of participants (children and adults) and adhesive types (self-curing and light-cured) showed no significant difference in bond failure rate between the direct and indirect bonding groups either. For sensitivity analyses, no significance of bond failure rate was found when the study of Aguirre et al was excluded (RR = 1.17, 95% CI =0.76–1.79, I^2^ = 36%, *P* = 0.47, or when odds ratio (OR = 1.15, 95% CI = 0.77–1.71, I^2^ = 23%, *P* = 0.51) was calculated (Additional file 2: Figure S2). Since less than 10 studies were included in the quantitative analysis on the bonding failure rate, the reporting bias was not assessed.

## Discussion

This study systematically reviewed and compared effectiveness (bracket placement accuracy), efficiency (total working time and chairside time) and adverse effects (oral hygiene status and bond failure rate) between direct and indirect bonding techniques for bonding orthodontic brackets and found that there was no significant difference between the two bonding techniques in bracket placement accuracy, oral hygiene status and bond failure rate. The total working time for indirect bonding was significantly longer than that for direct bonding, but the indirect bonding involved significantly less clinical chairside time than the direct bonding.

### Bracket placement accuracy

Accurate bracket positioning is of critical significance. The indirect bonding is believed to provide an accurate bracket positioning, because of unimpaired visibility and enough time, especially for the posterior teeth [29]. However, based on the current systematic review, there was no significant difference in the accuracy of bracket placement between the direct and indirect bonding techniques. This was explained by the assumption that brackets on the models might not be perfectly transferred to the patient’s dentition in the indirect bonding. Contaminants or soft tissue interferences, different thickness of bonding material in the clinical stage from that in the laboratory stage, errors in operations might result in the inaccurate transformation [30]. Zachrisson et al attached significant importance to clinical experience and indicated that orthodontic brackets could be precisely bonded using direct bonding technique by the experienced clinician after carefully studying plaster casts in advance. And a bracket-positioning gauge could be considered for the improvement of bracket placement accuracy [18]. The results disagreed with those of studies of Shpack et al. [8] or Koo et al. [31]. The latter two studies indicated the indirect bonding technique provided more accurate placement than the direct bonding. This inconsistency could be attributed to the less risk of contaminants or soft tissue interferences in the latter in vitro studies.

### Total working time and chairside time

In comparison with direct bonding, the indirect bonding was found to involve more time for the overall bonding process but less chairside time during the clinical phase according to the review. This was because the indirect bonding required a significant amount of time for the laboratorial phase [27] and saved the clinical chairside time by allowing several brackets to be bonded simultaneously [12, 27]. It was important to note that the total treatment time as well as the number of appointments had not been found to be significantly different between the direct and indirect bonding techniques [27]. This seemed to be consistent with another retrospective study which found the number of appointments and total treatment time showed no significant difference between the two techniques [29].

### Oral hygiene status

Prevention of poor oral hygiene and enamel demineralization is a great challenge faced by orthodontic clinicians. Several previous systematic reviews [32–36] focused the effect of bracket ligation on the oral hygiene and periodontal status, and found self-ligating brackets had no advantage over conventional brackets. Professional patient education and mechanical tooth cleaning were certificated to maintain good oral hygiene by two recent systematic reviews [37, 38]. Applying fluoride had been identified as the most useful intervention for orthodontically induced white spot lesions [39]. This systematic review discussed the effect of bonding techniques on oral hygiene status. Although one of the included studies [12] reported that the formation of biofilms and white spot lesions was greater in the direct bonding group than that in the indirect bonding during the first four months of treatment, no significant difference of oral hygiene status was found in the long-term follow-up (i.e. two and a half years) between the direct and indirect bonding techniques.

### Bond failure rate

No significant difference of bond failure rate was found between the direct and indirect bonding in the meta-analysis. This finding corroborated the idea of the in vivo part of a recent research [40] indicating that average survival rate for directly bonded brackets and indirectly bonded brackets was 98.6 and 98.3%, respectively, without significant difference. During the indirect bonding, it was often practically difficult to place a tray for multiple teeth correctly with a uniform and steady pressure on each tooth [41], which might result in uneven/excessive adhesives or low bonding strength [2, 18, 42]. But the moisture isolation, especially for posterior teeth, had been found to be relatively better in the indirect bonding due to the close-fitting transfer tray, which might provide a lower bond failure rate [30, 32, 33]. Whereas the working area in the oral cavity during the direct bonding remained visible, which made it possible to check the occlusal interference in real time [18].

There were variations in the age of participants, type of adhesives, and duration of trials in the literature. Some studies had reported that the adult patients had a lower failure rate than child patients [43], some studies on the other hand found there was no relation between the age and bond failure rate [2]. The subgroup analysis on the age of participants in the current study indicated that there was no significant difference between the two bonding techniques. The chemically-cured adhesive had been considered to polymerize once the two components were mixed and influenced the bonding failure rate, especially for the indirect bonding because the material loaded on the bracket bases in advance could result in polymerization out of sync and increased air inclusions in the bonding interface and layer [2, 25]. However, the subgroup analysis on the type of adhesives in the meta-analysis found that neither chemically cured nor light-cured had significant difference in the bonding failure rates of the two bonding techniques. An observation period for at least six months had been considered to be appropriate for assessing bonding failure rate [25]. All the recruited studies estimated the bonding effect in six months or longer except the study of Aguirre et al, in which they finished the trial in three months. Thus, a sensitivity analysis was conducted by excluding the study of Aguirre et al, and a consistent result was observed, indicating the stability of the meta-analysis.

### Others

The orthodontic brackets used in the included studies were all traditional non-customized buccal brackets. Though there has been an increasingly demand for the customized lingual fixed appliances due to their aesthetics [44], they are usually bonded with the indirect bonding technique because of the practical difficulty of placing lingual brackets accurately with the direct bonding technique, thus no comparison between direct and indirect bonding techniques was made when using customized lingual brackets in the study. A recent study compared the customized (Insignia) and non-customized (Damon Q) orthodontic systems and found that the customized group had more loose brackets, a longer planning time, and more complaints than the non-customized group [45]. More studies are needed in order to perform a systematic review or meta-analysis on the comparison of non-customized and customized systems (e.g. Incognito lingual brackets and Insignia labial brackets). In addition, it is recommended that clinicians should also take into account the other factors, such as clinical experience required for bracket positioning [46–48] and patient’s comfort [47], when choosing different bonding techniques in practice.

One limitation of the current study was the limited number of primary trials (i.e. 8 studies) and participants (i.e. 247 patients) included. High-quality and well-designed randomized controlled trials are needed. Based on the study, we recommend the future studies to use split-mouth design, set an observation period longer than 6 months, include the customized bracket systems, register the research protocol before performing the trial, and report the method used for randomization, blinding and concealment of allocation.

## Conclusions

Within the limitation of this systematic review, clinical evidence suggested that the direct and indirect bonding techniques had no significant difference in bracket placement accuracy, oral hygiene status and bond failure rate for bonding non-customized labial/buccal orthodontic brackets. The indirect bonding might require less chairside time but more total working time in comparison with the direct bonding technique. High-quality and well-designed randomized controlled trials are needed in order to make a conclusive recommendation.

## Additional files

Additional file 1: **Figure S1.** Forest plot for the risk ratio of bonding failure rate based on the age of participants (adults and children) and adhesive types (self-curing and light cured). (PDF 355 kb)

Additional file 2: **Figure S2.** Forest plot of sensitivity analyses. (PDF 218 kb)

Additional file 3: **Table S1.** PRSMA 2009 Checklist. (DOCX 19 kb)

Additional file 4: **Table S2.** Articles excluded from the systematic review based on the predetermined eligibility criteria. (DOCX 18 kb)

Additional file 5: **Table S3.** Risk of bias table. (DOCX 26 kb)

## Data Availability

All data generated or analyzed during this study are included in this published article and its supplementary information files

## Abbreviations

95% CI: 95% confidence interval
OR: Odds ratio
PRISMA: Preferred Reporting Items for Systematic Reviews and Meta-Analyses
RCTs: Randomized controlled trials
RR: Risk ratio
