# Supplementary figures and images for: Effectiveness, efficiency and adverse effects of using direct or indirect bonding technique in orthodontic patients: a systematic review and meta-analysis
Source: BMC Oral Health. 2019 Jul 8;19:137. doi: 10.1186/s12903-019-0831-4 (PMC6615229; doi:10.1186/s12903-019-0831-4)

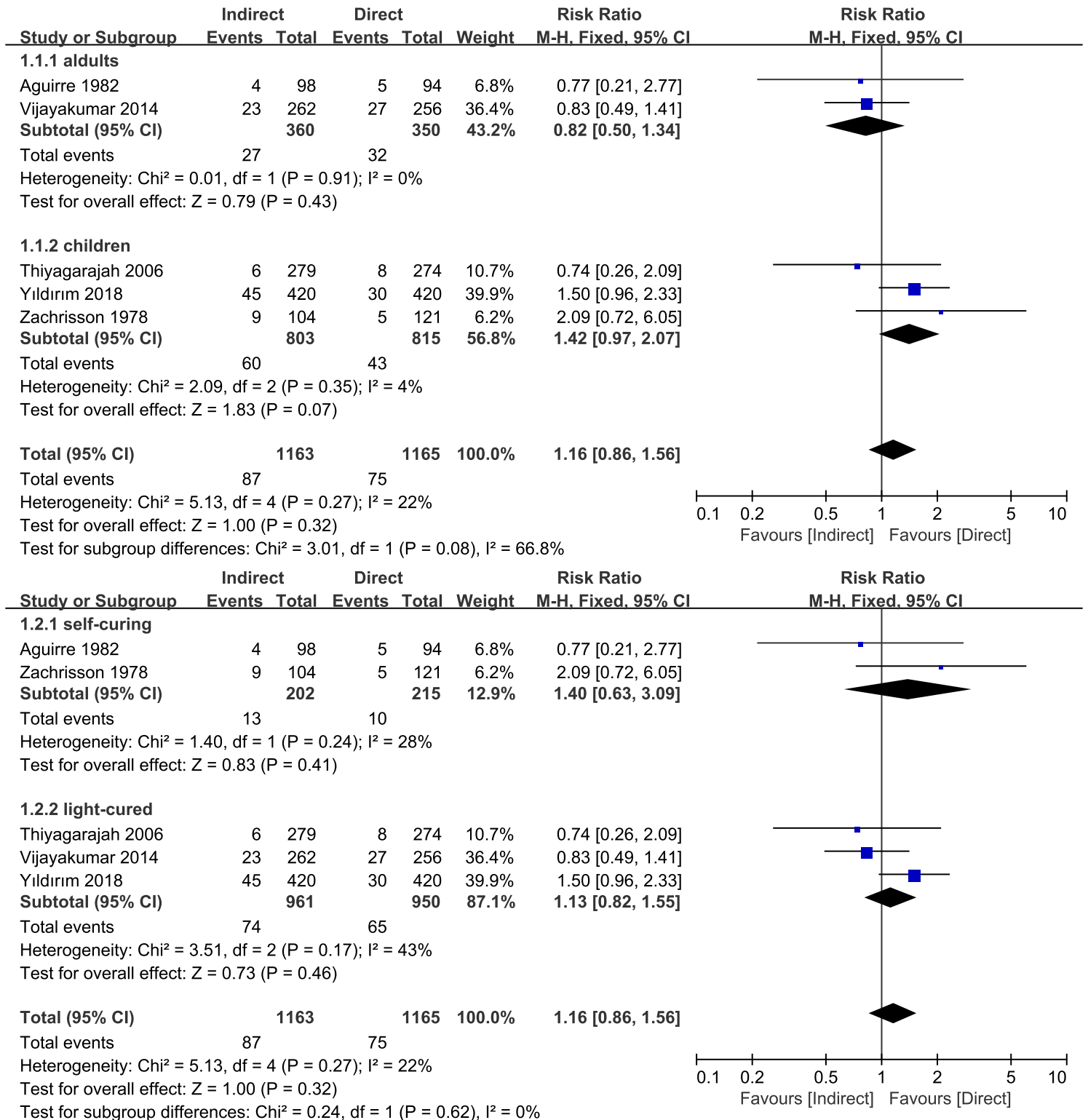

Supplement: Supplementary file 1 — Figure S1. Forest plot for the risk ratio of bonding failure rate based on the age of participants (adults and children) and adhesive types (self-curing and light cured). (PDF 355 kb) [file 12903_2019_831_MOESM1_ESM.pdf]

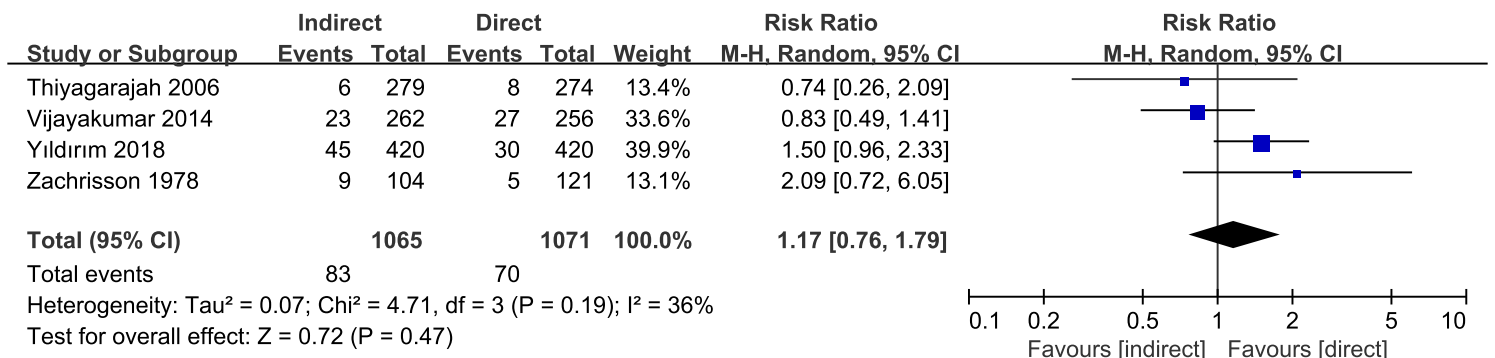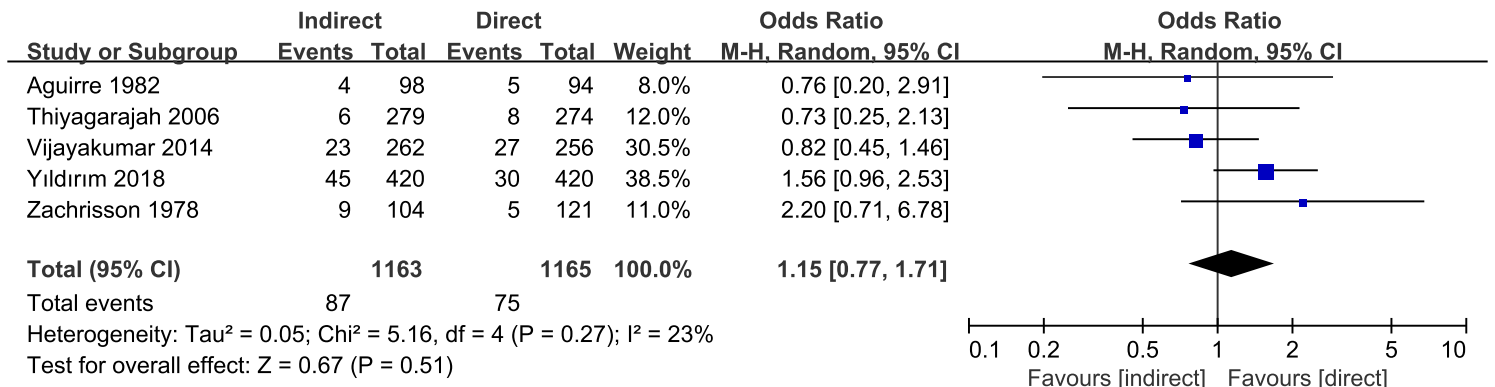

Supplement: Supplementary file 2 — Figure S2. Forest plot of sensitivity analyses. (PDF 218 kb) [file 12903_2019_831_MOESM2_ESM.pdf]
