# Supplementary material for: Effectiveness, efficiency and adverse effects of using direct or indirect bonding technique in orthodontic patients: a systematic review and meta-analysis
Source: BMC Oral Health. 2019 Jul 8;19:137. doi: 10.1186/s12903-019-0831-4 (PMC6615229; doi:10.1186/s12903-019-0831-4)
Supplement: Supplementary file 4 — Table S2. Articles excluded from the systematic review based on the predetermined eligibility criteria. (DOCX 18 kb) [file 12903_2019_831_MOESM4_ESM.docx]

**Table S2. Articles excluded from the systematic review based on the predetermined eligibility criteria**

| **Reason for exclusion** | **Studies** |
| --- | --- |
| Not comparing the bond failure | (1) |
| Review article | (2) |
| Study *in vitro* | (3-28) |
| Cross-sectional study | (29) |
| Technical report | (30) |
| Comment  Retrospective study | (31)  (32) |
| Prospective cohort study | (33, 34) |

1. Muguruma T, Yasuda Y, Iijima M, Kohda N, Mizoguchi I. Force and amount of resin composite paste used in direct and indirect bonding. Angle Orthod. 2010;80(6):1089-94.

2. Pellan P. Indirect bonding of brackets: don't wait another day! Int J Orthod. 2007;18(3):11-7.

3. Yi GK, Dunn WJ, Taloumis LJ. Shear bond strength comparison between direct and indirect bonded orthodontic brackets. Am J Orthod Dentofacial Orthop. 2003;124(5):577-81.

4. Yagci A, Uysal T, Ulker M, Ramoglu SI. Microleakage under orthodontic brackets bonded with the custom base indirect bonding technique. Eur J Orthod. 2010;32(3):259-63.

5. Yagci A, Uysal T, Ertas H, Amasyali M. Microleakage between composite/wire and composite/enamel interfaces of flexible spiral wire retainers: direct versus indirect application methods. Orthod Craniofac Res. 2010;13(2):118-24.

6. Wendl B, Droschl H, Muchitsch P. Indirect bonding - a new transfer method. Eur J Orthod. 2008;30(1):100-7.

7. Tortamano A, Nauff F, Naccarato SRF, Vigorito JW. Avaliação da força de tração em braquetes colados pela técnica indireta com diferentes sistemas de adesão. Revista Dental Press de Ortodontia e Ortopedia Facial. 2007;12(3):104-10.

8. Swetha M, Pai VS, Sanjay N, Nandini S. Indirect versus direct bonding--a shear bond strength comparison: an in vitro study. J Contemp Dent Pract 2011;12(4):232-8.

9. Shpack N, Geron S, Floris I, Davidovitch M, Brosh T, Vardimon AD. Bracket placement in lingual vs labial systems and direct vs indirect bonding. Angle Orthod. 2007;77(3):509-17.

10. Shimizu RH, Grando KG, Shimizu IA, Andriguetto AR, Melo ACM, Witters EL. Assessment of shear bond strength of brackets bonded by direct and indirect techniques: an in vitro study. Dental Press J Orthod. 2012;17(4):1-7.

11. Polat O, Karaman AI, Buyukyilmaz T. In vitro evaluation of shear bond strengths and in vivo analysis of bond survival of indirect-bonding resins. Angle Orthod. 2004;74(3):405-9.

12. Pamukcu H, Ozsoy OP, Dagalp R. In vitro and in vivo Comparison of Orthodontic Indirect Bonding Resins: A Prospective Study. Niger J Clin Pract. 2018;21(5):614-23.

13. Ozturk F, Ersoz M, Ozturk SA, Hatunoglu E, Malkoc S. Micro-CT evaluation of microleakage under orthodontic ceramic brackets bonded with different bonding techniques and adhesives. Eur J Orthod. 2016;38(2):163-9.

14. Ozturk F, Babacan H, Nalcaci R, Kustarci A. Effects of direct and indirect bonding techniques on bond strength and microleakage after thermocycling. Korean J Orthod. 2009;39(6):393-401.

15. Milne JW, Andreasen GF, Jakobsen JR. Bond strength comparison - a simplified indirect technique versus direct placement of brackets. Am J Orthod Dentofacial Orthop. 1989;96(1):8-15.

16. Lombardo L, Kaplan A, Lapenta R, Bratti E, Pera C, Scuzzo G, et al. A comparative study of lingual bracket bond strength. Orthodontics (CHIC). 2011;12(3):178-87.

17. Linn BJ, Berzins DW, Dhuru VB, Bradley TG. A comparison of bond strength between direct- and indirect-bonding methods. Angle Orthod. 2006;76(2):289-94.

18. Kwon T-Y, Meina H, Antoszewska J, Park H-S. Direct and indirect bonding of wire retainers to bovine enamel using three resin systems: shear bond strength comparisons. Korean J Orthod. 2011;41(6):447-53.

19. Koo BC, Chung CH, Vanarsdall RL. Comparison of the accuracy of bracket placement between direct and indirect bonding techniques. Am J Orthod Dentofacial Orthop. 1999;116(3):346-51.

20. Klocke A, Shi JM, Kahl-Nieke B, Bismayer U. Bond strength with custom base indirect bonding techniques. Angle Orthodontist. 2003;73(2):176-80.

21. Hocevar RA, Vincent HF. Indirect versus direct bonding - bond strength and failure location. Am J Orthod Dentofacial Orthop. 1988;94(5):367-71.

22. Flores T, Mayoral JR, Giner L, Puigdollers A. Comparison of enamel-bracket bond strength using direct- and indirect-bonding techniques with a self-etching ion releasing S-PRG filler. Dent Mater J. 2015;34(1):41-7.

23. Daub J, Berzins DW, Linn BJ, Bradley TG. Bond strength of direct and indirect bonded brackets after thermocycling. Angle Orthod. 2006;76(2):295-300.

24. Sifakakis I, Pandis N, Makou M, Katsaros C, Eliades T, Bourauel C. A comparative assessment of forces and moments generated by lingual and conventional brackets. Eur J Orthod. 2013;35(1):82-6.

25. Sinha PK, Nanda RS, Duncanson MG, Hosier MJ. Bond strengths and remnant adhesives resin on debonding for orthodontic bonding techniques. Am J Orthod Dentofacial Orthop. 1995;108(3):302-7.

26. Sinha PK, Nanda RS. The effect of different bonding and debonding techniques on debonding ceramic orthodontic brackets. Am J Orthod Dentofacial Orthop. 1997;112(2):132-7.

27. Sinha PK, Rohrer MD, Nanda RS, Brickman CD. Interlayer formation and its effect on debonding polycrystalline alumina orthodontic brackets. Am J Orthod Dentofacial Orthop. 1995;108(5):455-63.

28. Joseph VP, Rossouw PE, Basson NJ. SOME SEALANTS SEAL - A SCANNING ELECTRON-MICROSCOPY (SEM) INVESTIGATION. Am J Orthod Dentofacial Orthop. 1994;105(4):362-8.

29. Deahl ST, Salome N, Hatch JP, Rugh JD. Practice-based comparison of direct and indirect bonding. Am J Orthod Dentofacial Orthop. 2007;132(6):738-42.

30. Carlson SK, Johnson E. Bracket positioning and resets: Five steps to align crowns and roots consistently. Am J Orthod Dentofacial Orthop. 2001;119(1):76-80.

31. Benson P. Commentaries. A clinical comparison of bracket bond failures in association with direct and indirect bonding. J Orthod. 2006;33(3):185-.

32. Brown MW, Koroluk L, Ko C-C, Zhang K, Chen M, Nguyen T. Effectiveness and efficiency of a CAD/CAM orthodontic bracket system. Am J Orthod Dentofacial Orthop. 2015;148(6):1067-74.

33. Menini A, Cozzani M, Sfondrini MF, Scribante A, Cozzani P, Gandini P. A 15-month evaluation of bond failures of orthodontic brackets bonded with direct versus indirect bonding technique: a clinical trial. Prog Orthod. 2014;15:70.

34. Bozelli JV, Bigliazzi R, Barbosa HA, Ortolani CL, Bertoz FA, Faltin Junior K. Comparative study on direct and indirect bracket bonding techniques regarding time length and bracket detachment. Dental Press J Orthod. 2013;18(6):51-7.
