# Supplementary material for: Effectiveness, efficiency and adverse effects of using direct or indirect bonding technique in orthodontic patients: a systematic review and meta-analysis
Source: BMC Oral Health. 2019 Jul 8;19:137. doi: 10.1186/s12903-019-0831-4 (PMC6615229; doi:10.1186/s12903-019-0831-4)
Supplement: Supplementary file 5 — Table S3. Risk of bias table. (DOCX 26 kb) [file 12903_2019_831_MOESM5_ESM.docx]

**Table S3.** **Risk of bias table**

| **Study** | **Random**  **sequence generation** | **Allocation concealment** | **Blinding of participants and personnel** | **Blinding of outcome assessment** | **Incomplete outcome data** | **Selective reporting** | **Other bias** |
| --- | --- | --- | --- | --- | --- | --- | --- |
| Aguirre 1982 | Low risk:  **Quote:** “With the left side bonded by one technique and the right side by the other, as determined by the flip of a coin”  **Comment:** it was possibly done | Unclear risk:  The method to conceal the allocation was not mentioned; authors were emailed for details but there was no response | Unclear risk:  The blinding of participants and personnel was not mentioned; and it was unclear if non-blinding could affect the outcomes | Low risk:  The blinding of outcome assessment was not mentioned; but the outcome measurement was not likely to be influenced by lack of blinding | Low risk:  There were no dropouts during the trial | Low risk:  The protocol was not registered, but the predefined outcomes mentioned in the methods section seemed to have been reported | Low risk:  No other forms of bias seemed to be found |
| Dalessandri 2012 | Low risk:  **Quote:** “Randomization was performed by alphabetically listing patients and progressively assigning them a number between 1 and 30, then using a randomization table to rearrange the numbers into two columns, representing the two possible bonding procedures:”  **Comment:** it was possibly done | Unclear risk:  The method to conceal the allocation was not mentioned; authors were emailed for details but there was no response | Unclear risk:  **Quote:** “patients were treated by an orthodontist who was blinded to the study aims”  **Comment:**  The operator was blinded, but the blinding of participants was not mentioned; and it was unclear if the outcomes were affected | Low risk:  **Quote:** “A third operator used analysis of variance to statistically evaluate the data and was blinded regarding the origins of the measurements provenience.”  **Comment:** it was possibly done | Low risk:  There were no dropouts during the trial | Low risk:  The protocol was not registered, but the predefined outcomes mentioned in the methods section seemed to have been reported | Low risk:  No other forms of bias seemed to be found |
| Hodge 2004 | Unclear risk:  The method to generate random sequence was not mentioned; authors were emailed for details but there was no response | Unclear risk:  The method to conceal the allocation was not mentioned; authors were emailed for details but there was no response | Unclear risk:  The blinding of participants and personnel was not mentioned; and it was unclear if non-blinding could affect the outcomes | Unclear risk:  The blinding of outcome assessment was not mentioned; and it was unclear if non-blinding could affect the outcomes | Low risk:  There were no dropouts during the trial | Low risk:  The protocol was not registered, but the predefined outcomes mentioned in the methods section seemed to have been reported | Low risk:  No other forms of bias seemed to be found |
| Huang 2016 | Low risk:  **Quote:** “patients randomly assigned to three groups”  **Comment:** it was possibly done | Unclear risk:  The method to conceal the allocation was not mentioned; authors were emailed for details but there was no response | Unclear risk:  The blinding of participants and personnel was not mentioned; and it was unclear if non-blinding could affect the outcomes | Low risk:  The blinding of outcome assessment  was not mentioned; but the outcome measurement was not likely to be influenced by lack of blinding | Low risk:  There were no dropouts during the trial | Low risk:  The protocol was not registered, but the predefined outcomes mentioned in the methods section seemed to have been reported | Low risk:  No other forms of bias seemed to be found |
| Thiyagarajah 2006 | Low risk:  **Quote:** “Allocation to a group was made randomly”  **Comment:** it was possibly done | Unclear risk:  The method to conceal the allocation was not mentioned; authors were emailed for details but there was no response | Unclear risk:  The blinding of participants and personnel was not mentioned; and it was unclear if non-blinding could affect the outcomes | Low risk:  The blinding of outcome assessment  was not mentioned; but the outcome measurement was not likely to be influenced by lack of blinding | Low risk:  **Quote:** “Seven of the brackets placed indirectly required rebonding at the time of placement and these were not included in the results”  **Comment:** incomplete outcome data was adequately addressed. | Low risk:  The protocol was not registered, but the predefined outcomes mentioned in the methods section seemed to have been reported | Low risk:  No other forms of bias seemed to be found |
| Vijayakumar 2014 | Low risk:  **Quote:** “The patients  were randomly divided into two groups: Group A and Group B. A split-mouth design was used to randomize the study sample”  **Comment:** it was possibly done | Unclear risk:  The method to conceal the allocation was not mentioned; authors were emailed for details but there was no response | Unclear risk:  The blinding of participants and personnel was not mentioned; and it was unclear if non-blinding could affect the outcomes | Low risk:  The blinding of outcome assessment  was not mentioned; but the outcome measurement was not likely to be influenced by lack of blinding | Low risk:  There were no dropouts during the trial | Low risk:  The protocol was not registered, but the predefined outcomes mentioned in the methods section seemed to have been reported | Low risk:  No other forms of bias seemed to be found |
| Yıldırım 2018 | Low risk:  **Quote:** “The randomization sequence was created by a statistician who was not taking part in the study using an online randomization software (https://www.sealed envelope.com). The patients were allocated to treatment by block randomization in blocks of 4 with a 1:1 allocation ratio”  **Comment:** it was possibly done | Low risk:  **Quote:** “To secure the allocation concealment, the sequence generator was contacted by phone for group assignment after a patient was enrolled”  **Comment:** it was possibly done | Unclear risk:  **Quote:** “Neither the clinicians nor the patients were blinded to the intervention. ”  **Comment:** It was unclear if non-blinding could affect the outcomes | Low risk:  **Quote:** “The outcome assessor (K.Y.) was blinded during the dental cast and radiographic evaluations, data entry, and data analysis.” **Comment:** it was possibly done | Low risk:  There were no dropouts during the trial | Low risk:  The protocol was not registered, but the predefined outcomes mentioned in the methods section seemed to have been reported | Low risk:  No other forms of bias seemed to be found |
| Zachrisson 1978 | Low risk:  **Quote:** “Four different combinations of bonding techniques, adhesives, and bracket bases were tested in a randomized manner in maxillary and mandibular quadrants”  **Comment:** it was possibly done | Unclear risk:  The method to conceal the allocation was not mentioned; authors were emailed for details but there was no response | Unclear risk:  The blinding of participants and personnel was not mentioned; and it was unclear if non-blinding could affect the outcomes | Low risk:  Scoring of plaque accumulation and gingival condition was done by a blinded dental hygienist; although the blinding of bond failure rate assessor was not mentioned; the outcome was not likely to be influenced | Low risk:  There were no dropouts during the trial | Low risk:  The protocol was not registered, but the predefined outcomes mentioned in the methods section seemed to have been reported | Low risk:  No other forms of bias seemed to be found |
